# Identification of Predictors for Clinical Deterioration in Patients With COVID-19 via Electronic Nursing Records: Retrospective Observational Study

**DOI:** 10.2196/53343

**Published:** 2024-03-29

**Authors:** Sumi Sung, Youlim Kim, Su Hwan Kim, Hyesil Jung

**Affiliations:** 1 Department of Nursing Science, Research Institute of Nursing Science Chungbuk National University Cheongju, Chungcheongbuk-do Republic of Korea; 2 Department of Radiation Oncology College of Medicine Seoul National University Seoul Republic of Korea; 3 Department of Information Statistics Gyeongsang National University Jinju, Gyeongsangnam-do Republic of Korea; 4 Department of Nursing College of Medicine Inha University Incheon Republic of Korea

**Keywords:** COVID-19, infectious, respiratory, SARS-CoV-2, nursing records, SNOMED CT, random forest, logistic regression, EHR, EHRs, machine learning, documentation, deterioration, health records, health record, patient record, patient records, nursing, standardization, standard, standards, standardized, standardize, nomenclature, term, terms, terminologies, terminology

## Abstract

**Background:**

Few studies have used standardized nursing records with Systematized Nomenclature of Medicine–Clinical Terms (SNOMED CT) to identify predictors of clinical deterioration.

**Objective:**

This study aims to standardize the nursing documentation records of patients with COVID-19 using SNOMED CT and identify predictive factors of clinical deterioration in patients with COVID-19 via standardized nursing records.

**Methods:**

In this study, 57,558 nursing statements from 226 patients with COVID-19 were analyzed. Among these, 45,852 statements were from 207 patients in the stable (control) group and 11,706 from 19 patients in the exacerbated (case) group who were transferred to the intensive care unit within 7 days. The data were collected between December 2019 and June 2022. These nursing statements were standardized using the SNOMED CT International Edition released on November 30, 2022. The 260 unique nursing statements that accounted for the top 90% of 57,558 statements were selected as the mapping source and mapped into SNOMED CT concepts based on their meaning by 2 experts with more than 5 years of SNOMED CT mapping experience. To identify the main features of nursing statements associated with the exacerbation of patient condition, random forest algorithms were used, and optimal hyperparameters were selected for nursing problems or outcomes and nursing procedure–related statements. Additionally, logistic regression analysis was conducted to identify features that determine clinical deterioration in patients with COVID-19.

**Results:**

All nursing statements were semantically mapped to SNOMED CT concepts for “clinical finding,” “situation with explicit context,” and “procedure” hierarchies. The interrater reliability of the mapping results was 87.7%. The most important features calculated by random forest were “oxygen saturation below reference range,” “dyspnea,” “tachypnea,” and “cough” in “clinical finding,” and “oxygen therapy,” “pulse oximetry monitoring,” “temperature taking,” “notification of physician,” and “education about isolation for infection control” in “procedure.” Among these, “dyspnea” and “inadequate food diet” in “clinical finding” increased clinical deterioration risk (dyspnea: odds ratio [OR] 5.99, 95% CI 2.25-20.29; inadequate food diet: OR 10.0, 95% CI 2.71-40.84), and “oxygen therapy” and “notification of physician” in “procedure” also increased the risk of clinical deterioration in patients with COVID-19 (oxygen therapy: OR 1.89, 95% CI 1.25-3.05; notification of physician: OR 1.72, 95% CI 1.02-2.97).

**Conclusions:**

The study used SNOMED CT to express and standardize nursing statements. Further, it revealed the importance of standardized nursing records as predictive variables for clinical deterioration in patients.

## Introduction

As of September 27, 2023, the World Health Organization reported more than 770 million confirmed cases of COVID-19, including approximately 6.9 million deaths [1]. In South Korea, among 34,436,586 confirmed cases between January 3, 2020, and August 31, 2023, there were 35,812 deaths attributed to COVID-19 [2]. Among them, older patients or those with underlying diseases or comorbidities died due to severe conditions during the beginning of COVID-19; however, other cases exhibited initially mild symptoms that gradually worsened, causing death. Therefore, early detection of aggravating factors and symptoms is crucial for timely intervention and treatment.

COVID-19 symptoms include fever, cough, sore throat, nasal congestion, malaise, headache or muscle pain, dehydration, pneumonia, sepsis, and shortness of breath [3]. COVID-19 mortality prediction relies on patient age, blood oxygen saturation, and body temperature [4]. Through a systematic evaluation and external validation of 22 prognostic models for COVID-19 [5], admission oxygen saturation on room air and age have been shown to be strong predictors of clinical deterioration and mortality, respectively, in adults hospitalized with COVID-19.

The electronic health records (EHR) system contains detailed information about symptoms, problems, and services or care provided to patients with COVID-19. Previous studies have used EHR data to predict the COVID-19 prognosis [6-8]. However, nursing documentation records, which account for a substantial portion of EHR data, are underused for research owing to their low quality of documentation [9,10]. Unlike other EHR data, nursing documentation lacks standardization than other data such as diagnoses, laboratory, and medication data. The common data model (CDM) of the Observational Medical Outcome Partnership (OMOP) is designed to standardize the structure and content of observational data from multiple sites for efficient analyses that can produce reliable evidence; it is rarely used to standardize nursing documentation. One study [11] standardized 6277 nursing statements data using Systematized Nomenclature of Medicine-Clinical Terms (SNOMED CT) and converted them into the OMOP CDM to develop a fall-prediction model along with other CDM data. SNOMED CT is the most comprehensive multilingual clinical terminology [12,13] used by more than 40 countries and 30,000 individuals or organizations. The SNOMED CT International Edition, published on April 30, 2023, includes 360,942 concepts, 1,595,980 descriptions (synonyms) of concepts, and 3,261,032 relationships between concepts. Despite these advancements, there is a lack of empirical studies analyzing patient outcomes using standardized nursing documentation or records with SNOMED CT.

The American Nurses Association in 2015 and 2018 position statements also recommended the use of SNOMED CT to code nursing assessments or outcomes to facilitate the interoperability of nursing data. Several studies have examined the content coverage of SNOMED CT in the nursing domain by mapping standardized nursing terminologies, such as the Nursing Intervention Classification, International Classification for Nursing Practice (ICNP), Clinical Care Classification System, North American Nursing Diagnosis Association-International, and Omaha system, into SNOMED CT [14-18]. Thoroddsen et al [19] described the nursing care of patients with COVID-19 using ICNP and SNOMED CT. SNOMED CT outperformed ICNP in representing COVID-19 diagnoses and interventions. In particular, SNOMED CT comprehensively covered nursing interventions for patients with COVID-19. However, prior research only evaluated its usability or content coverage in the nursing domain, with limited analysis of specific phenomena or problems. Therefore, analyzing specific phenomena or problems using standardized nursing documentation data with SNOMED CT is necessary.

This study aimed to (1) standardize the nursing documentation records of patients with COVID-19 using SNOMED CT and (2) identify predictive factors of clinical deterioration in patients with COVID-19 using standardized nursing records.

## Methods

### Study Design and Setting

This retrospective observational study used data extracted from a clinical data warehouse (CDW) at a tertiary hospital with 1782 beds in Seoul, South Korea. The hospital operated 2 general wards and 4 disaster intensive care units (DICU) to treat COVID-19.

### Outcomes

In this study, the clinical outcome is the patient’s clinical deterioration. We defined clinical deterioration as an event in which patients with COVID-19 were transferred to the ICU from general wards.

### Study Participants

In total, 460 patients with COVID-19 were admitted to 2 general wards for infectious disease treatment at the hospital between December 2019 and June 2022. Patients transferred from or to other general wards or ICUs other than the DICU were excluded. Among the 460 patients, 19 patients were transferred to the DICU because of their deteriorating clinical condition. The mean length of stay (LOS) in the general ward of the 441 patients who were not transferred to the DICU during hospitalization was 8.71 days. The mean and maximum LOS of the 19 patients transferred to the DICU were 3.11 and 7 days, respectively.

Considering LOS differences between the 2 groups, the final analysis of the study included 226 admitted patients with COVID-19 at 2 general wards who were discharged or transferred to the DICU within 7 days between December 2019 and June 2022. Among these, 207 patients who were discharged in a stable clinical condition from the hospital within 7 days were assigned to the control group. A total of 19 patients who were transferred to the DICU within 7 days because of their deteriorating clinical conditions were assigned to the case group.

### Data Sources

Using CDW at a study hospital, the general characteristics of patients including age, gender, and initial respiratory symptoms were extracted from initial nursing assessment records. The patient’s acuity level and nursing statements were extracted from the nursing records. A total of 57,558 nursing statements from 226 patients were extracted. Among these, 45,852 nursing statements were obtained from the nursing records of 207 patients in the control group. Of the remaining records, 11,706 nursing statements were obtained from 19 patients in the case group.

### Mapping to International Standard Terminology, SNOMED CT

Nursing statements were standardized by mapping them to SNOMED CT concepts. The 57,558 nursing statements consisted of 1776 unique nursing statements which were interface terminologies developed by Seoul National University Hospital. Among the 1776 unique nursing statements, 260 unique nursing statements which were recorded 50,867 in total (cumulative recorded rate of 90%) were selected as the mapping source. Among these 260 unique statements, the control group used 237 nursing statements, and the case group used 204 nursing statements. 

According to the mapping guide in the previous study [20], 2 experts with more than 5 years of SNOMED CT mapping experience performed the mapping. The scope of the map was defined to restrict mapping to the precoordinated SNOMED CT concept only. Korean nursing statements were translated into English, and search terms were chosen considering the clinical context. SNOMED CT concepts are organized into 19 top-level concepts or hierarchies under the root concept. Among them, the Clinical finding (finding) hierarchy contains concepts related to symptoms and disorders. The procedure hierarchy contains concepts related to activities performed in the provision of health care and regime or therapy. The Situation with explicit context hierarchy includes concepts that specifically define the context information of a clinical finding or procedure [21]. Therefore, the nursing statements in nursing problems or outcomes and diagnosis domains were mapped to concepts of “clinical finding” and “situation with explicit context” hierarchies, and those in the nursing interventions domain were mapped to concepts of “procedure” hierarchy within the SNOMED CT International edition, released on November 30, 2022. If a SNOMED CT concept semantically consistent with the nursing statement could not be found, it was mapped to broader precoordinated concepts.

The mapping results were classified according to the level of correspondence as 3 types: “exact map,” “narrow to broad map,” and “no map.” Mappings were categorized as follows: “exact map” when the meaning of the nursing statement matched an equivalent SNOMED CT concept, “narrow to broad map” when the meaning of the nursing statement matched a broader SNOMED CT concept, and “no map” when there was no broad match concept for the meaning of the nursing statement.

Internal validation of the final map was conducted by calculating interrater reliability. Two experts reviewed the mapping results. If the mapper and reviewer selected the same result, the map was deemed correct. If the maps differed, the results were evaluated via a group discussion, and one of them was selected.

### Statistical Analyses

The general characteristics of patients (eg, gender, age, and patient acuity level) in both the case and control groups were explored. For patients in the case group transferred to the DICU (average ward LOS, 3.1 days), nursing statements documented up to the third day were extracted. The total number of nursing statements was divided by the LOS to calculate the mean number of nursing statements per day. Thereafter, the data set was separated according to the top-level hierarchies of SNOMED CT mapped to nursing statements, “clinical finding” or “situation with explicit context,” and “procedure.” Among the data set, we excluded SNOMED CT concepts that do not describe the patient’s clinical problems, such as no breathlessness, free of symptoms, no sputum, and no cough. To identify key features of nursing statements associated with the exacerbation of patient condition, random forest algorithms were used, and optimal hyperparameters were selected with 2 data sets (“clinical finding” or “situation with explicit context” and “procedure”).

To assess the effects of the features identified by random forest algorithms on the exacerbation of patient conditions, we performed logistic regression. Patient DICU transfer status was the dependent variable, and the top 5 features from each hierarchy identified by random forest algorithms were independent variables at a significance level of α=.05. Considering the small sample size for binary logistic regression analysis, the model was fitted using a modified estimation procedure known as Firth correction [22]. R (version 4.2.2; R Foundation for Statistical Computing) was used for all analyses.

### Ethical Considerations

This study was approved by the Institutional Review Board of the Seoul National University Hospital (H-2207-097-1341). The requirement for informed consent was waived according to the relevant guidelines and regulations of the institutional review board. Identifiers, such as the patient’s ID and name, were encrypted so that individuals could not be identified during the data analysis. Participant compensation was not offered since this study was a retrospective observational study.

## Results

### SNOMED CT Mapping Results

The results of mapping nursing statements to SNOMED CT are presented in Table 1. The interrater reliability of the mapping results was excellent (87.7%). Of the 260 unique nursing statements, 157 were on nursing problems or outcomes, 16 were on nursing diagnoses, and 87 were on nursing intervention statements. A total of 138 (87.8%) of nursing problems or outcomes statements were mapped to concepts within “clinical finding” and “situation with explicit context” hierarchies on SNOMED CT. Through a validation process, 19 nursing problems and outcome statements were mapped into concepts within the procedure hierarchy. All 16 nursing diagnosis statements were mapped to clinical finding concepts. Among the 87 nursing intervention statements, 85 (97.7%) nursing intervention statements were mapped to concepts within the procedure hierarchy. Only 2 statements were mapped to the clinical finding concepts, “267038008 |Edema|” and “29658002 |Oxygen supply absent|.” Of the 260 nursing statements, 244 (93.8%) nursing statements were classified as “exact map” and 16 (6.2%) nursing statements were classified as “broad map.” The mapped concepts with high frequency are presented in Multimedia Appendix 1.

**Table 1:** Results of mapping nursing statements to Systematized Nomenclature of Medicine–Clinical Terms.

Type of statements and top-level hierarchy		Concepts, n^a^
**Nursing problems or outcomes (n=157 statements)**		
	Clinical finding	94
	Situation with explicit context	22
	Procedure	17
**Nursing diagnoses (n=16 statements)**		
	Clinical finding	15
**Nursing interventions (n=87 statements)**		
	Procedure	64
	Clinical finding	2
**Total (n=260 statements)**		
	Clinical finding	109
	Procedure	73
	Situation with explicit context	22

^a^The number of concepts was calculated after removing duplicated concepts.

### Clinical Characteristics

The clinical characteristics of the study participants are presented in Table 2. The mean age was 55.9 (SD 17.3) years, and the mean LOS was 5.3 (SD 2.0) days. Patients in the control group were hospitalized in the ward for approximately 5.04 (SD 1.6) days. Patients in the case group were admitted to the ward for approximately 3.1 (SD 1.9) days. Overall, 119 (52.7%) patients were male. A total of 197 (86.7%) patients had a patient acuity level of 4, and 26 (11.5%) patients had a patient acuity level of 3. Additionally, 59 (26.1%) patients had a cough, 47 (20.8%) patients had sputum, and 26 (11.5%) patients had dyspnea as their initial symptoms.

**Table 2:** Clinical characteristics of study participants.

Variables			Control group (n=207)		Case group (n=19)		Total (n=226)		Chi-square (*df*) or *t* test (*df*)			*P* value	
Age (years), mean (SD)			55.8 (17.3)		57.0 (18.6)		55.9 (17.3)		–0.28 (224)^a^			.78	
**Age (years), n (%)**										3.7 (5)^b^			.60
	<30	14 (6.8)		1 (5.3)		15 (6.6)							
	30-39	30 (14.5)		4 (21.1)		34 (15)							
	40-49	30 (14.5)		0 (0)		30 (13.3)							
	50-59	38 (18.4)		4 (21.1)		42 (18.6)							
	60-69	42 (20.3)		5 (26.3)		47 (20.8)							
	>70	53 (25.6)		5 (26.3)		58 (25.7)							
**Sex, n (%)**										0.1 (1)^b^			.81
	Male	110 (53.1)		9 (47.4)		119 (52.7)							
	Female	97 (46.9)		10 (52.6)		107 (47.3)							
**LOS^c^, mean (SD)**													
	LOS ward	5.04 (1.6)		3.1 (1.9)		4.9 (1.7)		5.06 (224)^a^			<.001		
	LOS DICU^d^	0.0 (0)		5 (2.9)		0.4 (1.6)		–7.50 (18)^a^			<.001		
	LOS total	5.04 (1.6)		8.1 (3.2)		5.3 (2)		–4.11 (18.882)^a^			<.001		
**Patient acuity level, n (%)**										8.4 (2)^b^			.01
	Level 1	0 (0)		0 (0)		0 (0)							
	Level 2	4 (1.9)		0 (0)		4 (1.8)							
	Level 3	20 (9.7)		6 (31.6)		26 (11.5)							
	Level 4	183 (88.4)		13 (68.4)		196 (86.7)							
**Initial respiratory symptoms, n (%)**													
	Cough	53 (25.6)		6 (31.6)		59 (26.1)		0.1 (1)^b^			.76		
	Sputum	40 (19.3)		7 (36.8)		47 (20.8)		2.3 (1)^b^			.13		
	Dyspnea	19 (9.2)		7 (36.8)		26 (11.5)		10.5 (1)^b^			<.001		
	Other	6 (2.9)		0 (0)		6 (2.7)		0.0 (1)^b^			.99		
	None	144 (55.1)		11 (57.9)		71 (31.4)		0.6 (1)^b^			.43		
**Nursing statements**													
	Frequency in total, n	45,852		11,706		57,558		N/A^e^			N/A		
	Frequency per day, per patient; mean (SD)	42.73 (14.60)		55.48 (22.01)		43.80 (15.70)		–2.48 (19.48)^a^			.023		

^a^*t* test.

^b^Chi-square.

^c^LOS: length of stay.

^d^DICU: disaster intensive care units.

^e^N/A: not applicable.

### Feature Selection via Random Forest

**Figure 1:**
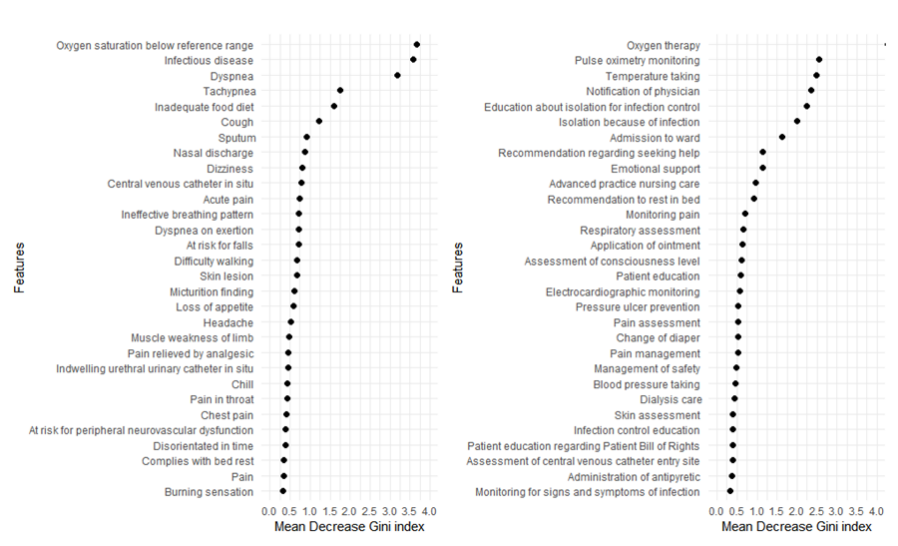
Top 30 features extracted via random forest (left: clinical finding or situation with explicit context concepts; right: procedure concepts).

The feature importance calculated using the random forest method is shown in Figure 1. The most important feature in “clinical finding” showed “449171008 |Oxygen saturation below reference range (finding)|.” Prominent concepts that express respiratory issues included “267036007 |Dyspnea (finding)|,” “271823003 |Tachypnea (finding)|,” and “49727002 |Cough (finding)|.”

Among procedure concepts, “57485005 |Oxygen therapy|” was the most important feature, followed by “284034009 |Pulse oximetry monitoring|,” “56342008 |Temperature taking (procedure)|,” “428426009 |Notification of physician|,” and “737612005 |Education about isolation for infection control|.”

### Association Between Patient’s DICU Transfer and Selected Features

Table 3 presents the results of logistic regression analysis, which explored the association between patient DICU transfer status and features in both “clinical finding” or “situation with explicit context” and “procedure” selected via random forest. In “clinical finding,” patients with dyspnea and inadequate food diet were more likely to experience DICU transfer (dyspnea: odds ratio [OR] 5.99, 95% CI 2.25-20.29; inadequate food diet: OR 10.0, 95% CI 2.71-40.84). Patients with infectious disease in nursing records were less likely to be associated with the DICU transfer in patients with COVID-19 (OR 0.32, 95% CI 0.16-0.66). In the procedure, patients with “oxygen therapy” and “notification of physician” were more likely to experience DICU transfer (oxygen therapy: OR 1.89, 95% CI 1.25-3.05; notification of physician: OR 1.72, 95% CI 1.02-2.97). Patients with “temperature taking” were less likely to experience DICU transfer (OR 0.36, 95% CI 0.18-0.70).

**Table 3:** Results of logistic regression.

SNOMED CT^a^ top-level hierarchy and SNOMED CT code		SNOMED CT fully specified name	Odds ratio (95% CI)	*P* values
**Clinical finding**				
	449171008	Oxygen saturation below reference range	4.21 (0.88-18.78)	.07
	40733004	Infectious disease	0.32 (0.16-0.66)	.003
	267036007	Dyspnea	5.99 (2.25-20.29)	<.001
	271823003	Tachypnea	2.22 (0.34-44.75)	.44
	102610002	Inadequate food diet	10.0 (2.71-40.84)	.001
**Procedure**				
	57485005	Oxygen therapy	1.89 (1.25-3.05)	.002
	284034009	Pulse oximetry monitoring	0.84 (0.46-1.51)	.55
	56342008	Temperature taking	0.36 (0.18-0.70)	.002
	428426009	Notification of physician	1.72 (1.02-2.97)	.04
	737612005	Education about isolation for infection control	1.26 (0.76-2.02)	.35

^a^SNOMED CT: Systematized Nomenclature of Medicine-Clinical Terms.

## Discussion

### Principal Results

This study standardized the nursing statements of patients with COVID-19 in a hospital in South Korea by mapping them to SNOMED CT, an international standard terminology. Using these standardized statements, machine learning analysis with a random forest was conducted. Concepts that could predict patient deterioration in nursing problems or outcomes and intervention domains were identified. In the nursing problems or outcomes domain, concepts in nursing statements related to dyspnea, tachypnea, and oxygen saturation below the reference range were associated with patient deterioration. In nursing interventions, concepts in nursing statements related to respiratory intervention, including oxygen therapy, pulse oximetry monitoring, and physician notification, were associated with DICU transfer. These concepts were different from the findings of another study that analyzed nursing statements of patients with ovarian cancer. Kim et al [23] analyzed standardized nursing statements of patients who underwent curative surgery for epithelial ovarian cancer, and urination, food supply, bowel mobility, and pain were identified as the most common concepts.

To the best of our knowledge, this study is the first to identify predictive factors from nursing records standardized using SNOMED CT. Although previous studies [24-29] have used standardized nursing records such as the North American Nursing Diagnosis Association-International, Nursing Intervention Classification, Nursing Outcomes Classification, and ICNP to predict the patient’s condition or explore the nursing care provided, no study has predicted patient clinical deterioration using SNOMED CT–standardized nursing records. To achieve interoperable health information exchange, the Centers for Medicare and Medicaid Services and Office of the National Coordinator for Health Information Technology recommended the use of SNOMED CT and Logical Observation Identifier Names and Codes (LOINC) as reference terminologies. In 2020, the Office of the National Coordinator for Health Information Technology introduced the United States Core Data for Interoperability, a standardized set of health data classes and constituent data elements that recommended the use of international standard terminologies, including SNOMED CT and LOINC [30]. Consequently, standardization of nursing records using reference terminology before conducting data analysis is required in the nursing field.

Our study comprehensively standardized all nursing statements in study participants using SNOMED CT concepts. Several previous studies have evaluated SNOMED CT coverage in various health care domains, such as frailty and wound care [31-34]. In nursing, efforts have been made to map ICNP 7-axis concepts, nursing problem lists, and ICU nursing flowsheets to SNOMED CT [16,32,35-37], which provides the most detailed semantic expression compared with other standard terminologies [19]. Notably, this study unexpectedly mapped 19 nursing statements on nursing assessments or outcomes to the “procedure” hierarchy, despite the lack of corresponding concepts in the “clinical finding” or “situation with explicit context” hierarchies. This was due to the fact that the problem or phenomenon was the result of specific nursing procedures or practices. For example, the statement “a patient is applying a mattress to prevent bedsores” in nursing assessment or outcome was mapped to concept “733920005 |Provision of pressure-relieving mattress|” in the procedure hierarchy.

The identified features affecting patient deterioration (DICU transfer) in the hierarchy of “clinical finding” included respiratory issues such as low oxygen saturation, dyspnea, and tachypnea. Izquierdo et al [7] reported patient signs and symptoms, especially tachypnea, to be reliable predictors of DICU admission, which is consistent with our results. In addition, inadequate diet was significantly associated with DICU transfer, potentially due to the inability of patients to eat independently owing to their deteriorated condition. These findings highlighted the importance of signs and symptoms in nursing records for predicting a patient’s deteriorating clinical condition. Alternatively, nursing statements related to “infectious disease” within “clinical finding” and “temperature taking” within “procedure” decreased the risk of clinical deterioration in patients with COVID-19. Since patients with COVID-19 were included as study participants, it was valid that the concept of “infectious disease” appeared frequently in nursing statements, but repeated studies are needed to explain the finding that the more statements related to this concept, the lower the risk of clinical deterioration. Considering previous studies [4,38] reporting “fever” as a significant factor affecting deterioration in patients with COVID-19, our study revealed that increased nursing activity of “temperature taking” notably reduced patient deterioration risk. This suggested that nurses may have preemptively frequently monitored temperature to prevent fever; however, to accurately interpret this result, analyzing “body temperature” data is necessary. The features identified in this study highlight the potential of nursing records as a valuable real time predictor of a patient’s clinical condition.

### Limitations

This study had some limitations. First, the analyzed data were extracted from a single tertiary university hospital, which limits the generalizability of this study. Second, sample bias is possible because patients admitted for more than 7 days to the ward were excluded. Third, other clinical findings or nursing interventions may not have been documented as nursing statements in the nursing records. Considering that this study extracted only structured nursing statements stored in the CDW, records written in free text were not included in the analysis. Fourth, as SNOMED CT concepts were used in the analysis, the results may vary depending on the hierarchical structure or level of the mapped SNOMED CT concept. For example, concepts such as “chest pain,” “headache,” and “pain” derived through random forest are connected in a hierarchical structure where “chest pain” and “headache” are subcategories of the “pain” concept. If “chest pain” and “headache” were grouped together as “pain,” the effect of “pain” on clinical deterioration might have been significant.

### Conclusions

This study showed that standardized nursing records are an important source of data that can be used to predict clinical deterioration in patients with COVID-19. In total, 260 nursing statements were mapped to the SNOMED CT, including 109 concepts in the clinical finding hierarchy, 73 concepts in the procedure hierarchy, and 22 concepts in “situation” with an explicit context hierarchy. Among the standardized nursing statements, key clinical findings were respiratory issues, including low oxygen saturation, dyspnea, and tachypnea. The primary procedure-related features included oxygen therapy, pulse oximetry, and temperature monitoring. In specific, low oxygen saturation, dyspnea, tachypnea, and oxygen therapy are associated with the risk of clinical deterioration in patients with COVID-19. This study validates the use of nursing records as variables for predicting the deterioration of patients with COVID-19. Future research should investigate the integration of standardized nursing records with diagnoses, laboratory, and medication data to develop a highly reliable predictive model.

## Appendix

Multimedia Appendix 1 The mapped concepts with high frequency.

## Abbreviations

CDM: common data model
CDW: clinical data warehouse
DICU: disaster intensive care unit
EHR: electronic health record
ICNP: International Classification for Nursing Practice
LOINC: Logical Observation Identifier Names and Codes
LOS: length of stay
NANDA-I: North American Nursing Diagnosis Association–International
NIC: Nursing Intervention Classification
OMOP: Observational Medical Outcome Partnership
ONC: Office of the National Coordinator for Health Information Technology
OR: odds ratio
SNOMED CT: Systematized Nomenclature of Medicine–Clinical Terms

## Data Availability

The data analyzed during this study are not readily available because of hospital regulation restrictions and patient privacy concerns. Requests to access the data sets should be directed to the corresponding author.

## References

[1] (2023). WHO Coronavirus (COVID-19) dashboard. World Health Organization.

[2] Coronavirus disease 19 (COVID-19) occurrence status. Korea Disease Control and Prevention Agency.

[3] (2020). Clinical management of severe acute respiratory infection when novel coronavirus (2019-nCoV) infection is suspected: interim guidance. World Health Organization.

[4] Yadaw AS, Li YC, Bose S, Iyengar R, Bunyavanich S, Pandey G (2020). Clinical features of COVID-19 mortality: development and validation of a clinical prediction model. Lancet Digit Health.

[5] Gupta RK, Marks M, Samuels THA, Luintel A, Rampling T, Chowdhury H, Quartagno M, Nair A, Lipman M, Abubakar I, van Smeden M, Wong WK, Williams B, Noursadeghi M (2020). Systematic evaluation and external validation of 22 prognostic models among hospitalised adults with COVID-19: an observational cohort study. Eur Respir J.

[6] Campbell TW, Wilson MP, Roder H, MaWhinney S, Georgantas RW, Maguire LK, Roder J, Erlandson KM (2021). Predicting prognosis in COVID-19 patients using machine learning and readily available clinical data. Int J Med Inform.

[7] Izquierdo JL, Ancochea J, Soriano JB, Savana COVID-19 Research Group (2020). Clinical characteristics and prognostic factors for intensive care unit admission of patients with COVID-19: retrospective study using machine learning and natural language processing. J Med Internet Res.

[8] Gong K, Wu D, Arru CD, Homayounieh F, Neumark N, Guan J, Buch V, Kim K, Bizzo BC, Ren H, Tak WY, Park SY, Lee YR, Kang MK, Park JG, Carriero A, Saba L, Masjedi M, Talari H, Babaei R, Mobin HK, Ebrahimian S, Guo N, Digumarthy SR, Dayan I, Kalra MK, Li Q (2021). A multi-center study of COVID-19 patient prognosis using deep learning-based CT image analysis and electronic health records. Eur J Radiol.

[9] Wang N, Hailey D, Yu P (2011). Quality of nursing documentation and approaches to its evaluation: a mixed-method systematic review. J Adv Nurs.

[10] Fennelly O, Grogan L, Reed A, Hardiker NR (2021). Use of standardized terminologies in clinical practice: a scoping review. Int J Med Inform.

[11] Jung H, Yoo S, Kim S, Heo E, Kim B, Lee HY, Hwang H (2022). Patient-level fall risk prediction using the observational medical outcomes partnership's common data model: pilot feasibility study. JMIR Med Inform.

[12] Lee D, de Keizer N, Lau F, Cornet R (2014). Literature review of SNOMED CT use. J Am Med Inform Assoc.

[13] Park HA, Yu SJ, Jung H (2021). Strategies for adopting and implementing SNOMED CT in Korea. Healthc Inform Res.

[14] Park HT, Lu DF, Konicek D, Delaney C (2007). Nursing interventions classification in systematized nomenclature of medicine clinical terms: a cross-mapping validation. Comput Inform Nurs.

[15] Park HA, Lundberg C, Coenen A, Konicek D (2011). Evaluation of the content coverage of SNOMED CT representing ICNP seven-axis version 1 concepts. Methods Inf Med.

[16] Kim TY, Coenen A, Hardiker N (2012). Semantic mappings and locality of nursing diagnostic concepts in UMLS. J Biomed Inform.

[17] Monsen KA, Finn RS, Fleming TE, Garner EJ, LaValla AJ, Riemer JG (2016). Rigor in electronic health record knowledge representation: lessons learned from a SNOMED CT clinical content encoding exercise. Inform Health Soc Care.

[18] Kim J, Yao Y, Macieira TGR, Keenan G (2019). An examination of the coverage of the SNOMED CT coded nursing problem list subset. JAMIA Open.

[19] Thoroddsen A, Rúnarsdóttir ER, Örlygsdóttir B (2023). Description of COVID-19 patients and mapping nursing data to ICNP 2021 reference set in SNOMED CT. Int Nurs Rev.

[20] Sung S, Park HA, Jung H, Kang H (2023). A SNOMED CT mapping guideline for the local terms used to document clinical findings and procedures in electronic medical records in South Korea: methodological study. JMIR Med Inform.

[21] (2023). SNOMED CT editorial guide. SNOMED CT International.

[22] van Smeden M, de Groot JAH, Moons KGM, Collins GS, Altman DG, Eijkemans MJC, Reitsma JB (2016). No rationale for 1 variable per 10 events criterion for binary logistic regression analysis. BMC Med Res Methodol.

[23] Kim K, Han Y, Jeong S, Doh K, Park HA, Lee K, Cho M, Ahn S (2019). Prediction of postoperative length of hospital stay based on differences in nursing narratives in elderly patients with epithelial ovarian cancer. Methods Inf Med.

[24] Azzolin K, Mussi CM, Ruschel KB, de Souza EN, de Fátima Lucena A, Rabelo-Silva ER (2013). Effectiveness of nursing interventions in heart failure patients in home care using NANDA-I, NIC, and NOC. Appl Nurs Res.

[25] Escalada-Hernández P, Muñoz-Hermoso P, González-Fraile E, Santos B, González-Vargas JA, Feria-Raposo I, Girón-García JL, García-Manso M (2015). A retrospective study of nursing diagnoses, outcomes, and interventions for patients with mental disorders. Appl Nurs Res.

[26] da Silva Thomé E, Centena RC, da Silva Behenck A, Marini M, Heldt E (2014). Applicability of the NANDA-I and nursing interventions classification taxonomies to mental health nursing practice. Int J Nurs Knowl.

[27] Yang MJ, Kim HY, Ko E, Kim HK (2019). Identification of nursing diagnosis-outcome-intervention linkages for inpatients in the obstetrics department nursing unit in South Korea. Int J Nurs Knowl.

[28] Palomar-Aumatell X, Subirana-Casacuberta M, Mila-Villarroel R (2017). Critical care nursing interventions and the time required for their completion in intensive care units: a Delphi study. Intensive Crit Care Nurs.

[29] Sung S, Jung H, Kim Y (2024). Exploring nursing care for patients with COVID-19 using international classification for nursing practice-based nursing records. Comput Inform Nurs.

[30] (2023). United States Core Data for Interoperability (USCDI). Office of the National Coordinator for Health Information Technology.

[31] Thandi M, Brown S, Wong ST (2021). Mapping frailty concepts to SNOMED CT. Int J Med Inform.

[32] Block LJ, Wong ST, Handfield S, Hart R, Currie LM (2021). Comparison of terminology mapping methods for nursing wound care knowledge representation. Int J Med Inform.

[33] Hüsers J, Przysucha M, Esdar M, John SM, Hübner UH (2021). Expressiveness of an international semantic standard for wound care: mapping a standardized item set for leg ulcers to the systematized nomenclature of medicine-clinical terms. JMIR Med Inform.

[34] Block L, Handfield S, Sermeus W, Procter PM, Weber P (2016). Mapping wound assessment data elements in SNOMED CT. Nursing Informatics 2016: eHealth for All: Every Level Collaboration—From Project to Realization.

[35] Matney SA, Warren JJ, Evans JL, Kim TY, Coenen A, Auld VA (2012). Development of the nursing problem list subset of SNOMED CT®. J Biomed Inform.

[36] So EY, Park HA (2011). Exploring the possibility of information sharing between the medical and nursing domains by mapping medical records to SNOMED CT and ICNP. Healthc Inform Res.

[37] Park HA, Lundberg CB, Coenen A, Konicek DJ, Saranto K, Brennan PF, Park HA, Tallberg M, Ensio A (2009). Evaluation of the content coverage of SNOMED-CT to represent ICNP version 1 catalogues. Connecting Health and Humans.

[38] Chen SL, Feng HY, Xu H, Huang SS, Sun JF, Zhou L, He JL, Song WL, Wang RJ, Li X, Fang M (2020). Patterns of deterioration in moderate patients with COVID-19 from jan 2020 to mar 2020: a multi-center, retrospective cohort study in China. Front Med (Lausanne).

