# Supplementary material for: Identification of Predictors for Clinical Deterioration in Patients With COVID-19 via Electronic Nursing Records: Retrospective Observational Study
Source: J Med Internet Res. 2024 Mar 29;26:e53343. doi: 10.2196/53343 (PMC10984341; doi:10.2196/53343)
Supplement: Multimedia Appendix 1 [file jmir_v26i1e53343_app1.docx]

| SNOMED CT  top-level hierarchy | SNOMED  CT ID | SNOMED CT Fully Specified Name | Records frequency in total,  N (%) | Records per day,  Mean (SD) |
| --- | --- | --- | --- | --- |
| Clinical finding/ Situation with explicit context | 40733004 | Infectious disease | 2717 (6.20) | 2.38 (0.52) |
|  | 162467007 | Free of symptoms | 2433 (5.55) | 2.12 (1.03) |
|  | 49727002 | Cough | 1651 (3.77) | 1.43 (1.05) |
|  | 161938003 | No breathlessness | 1348 (3.07) | 1.16 (1.05) |
|  | 248596009 | Sputum - symptom | 1336 (3.05) | 1.16 (1.09) |
|  | 274663001 | Acute pain | 1271 (2.90) | 1.14 (1.20) |
|  | 248602007 | No sputum | 1181 (2.69) | 1.04 (1.06) |
|  | 161922009 | No cough | 866 (1.98) | 0.79 (1.02) |
|  | 20573003 | Ineffective breathing pattern | 850 (1.94) | 0.70 (1.06) |
|  | 60845006 | Dyspnea on exertion | 594 (1.35) | 0.46 (0.89) |
| Procedure | 170497006 | Isolation because of infection | 2829 (6.45) | 2.53 (0.44) |
|  | 56342008 | Temperature taking | 2590 (5.91) | 2.24 (0.72) |
|  | 284034009 | Pulse oximetry monitoring | 2395 (5.46) | 2.09 (1.26) |
|  | 304979007 | Recommendation regarding seeking help | 1724 (3.93) | 1.46 (0.92) |
|  | 225399009 | Pain assessment | 1467 (3.35) | 1.30 (1.44) |
|  | 422834003 | Respiratory assessment | 1113 (2.54) | 0.94 (1.32) |
|  | 57485005 | Oxygen therapy | 1000 (2.28) | 0.89 (1.57) |
|  | 428426009 | Notification of physician | 791 (1.8) | 0.78 (0.88) |
|  | 737612005 | Education about isolation for infection control | 505 (1.15) | 0.53 (0.95) |
|  | 710995003 | Monitoring pain | 493 (1.12) | 0.47 (0.76) |

Supplementary table 1. The mapped concepts with high frequency
